# A Prospective Study Evaluating IOP Changes after Switching from a Therapy with Prostaglandin Eye Drops Containing Preservatives to Nonpreserved Tafluprost in Glaucoma Patients

**DOI:** 10.1100/2012/804730

**Published:** 2012-04-19

**Authors:** Stefano Ranno, Matteo Sacchi, Cinzia Brancato, Daniela Gilardi, Andrea Lembo, Paolo Nucci

**Affiliations:** ^1^Eye Clinic, San Giuseppe Hospital, University of Milan, Via San Vittore 12, 20123 Milan, Italy; ^2^Frontier Science & Technology Research Foundation Southern Europe (FSE), 6830 Chiasso, Switzerland; ^3^Data Management Service, Multimedica Group, 20159 Milan, Italy

## Abstract

*Purpose*. To compare the ocular hypotensive effect of tafluprost with prostaglandin analogues (PGAs) in glaucoma patients. *Methods*. 89 primary open-angle glaucoma patients treated with bimatoprost, latanoprost, or travoprost for at least 3 months complaining for ocular discomfort were switched to tafluprost. IOP was assessed at baseline and 3 months after switching the therapy by daily curve. Primary outcome was to compare the mean daily IOP of tafluprost with PGAs. *Results*. The mean daily IOP was 16 ± 2.1 and 16.6 ± 2.0 mm Hg at baseline and after switching to tafluprost, respectively (*P* > 0.05). When analysis was carried out between tafluprost and each previous PGAs, the comparison between latanoprost and tafluprost and travoprost and tafluprost did not show any statistically significant difference in mean daily IOP and at each time point. The comparison between bimatoprost and tafluprost showed a statistically significant difference in mean daily IOP (*P* < 0.05) and at each time point (*P* < 0.05). *Conclusions*. After 3 months of switching tafluprost showed an overall IOP lowering effect similar to others PGAs. When each PGA was compared with tafluprost, bimatoprost showed to provide a statistically significant additional IOP lowering effect.

## 1. Introduction

Intraocular pressure (IOP) is known to be the main risk factor for development and progression of glaucoma [1, 2]. Lowering intraocular pressure is the only evidence-based method for treating glaucoma, reducing the risk of visual field progression from 13% to 19% per 1 mmHg of IOP lowering [3, 4].

According to the European Glaucoma Society Guidelines, topical monotherapy is the first step in the medical management [1].

Among the many topical hypotensive medications, prostaglandin analogues (PGAs) are proved to be the most potent in lowering IOP and with very few systemic side effects [5, 6].

PGAs were first proposed for glaucoma treatment by Camras et al. in 1977 [7].  Nowadays, derivatives of prostaglandin F_2*α*_, that is, latanoprost, travoprost, unoprostone, prostamide, and bimatoprost are commercially available. In van der Valk's meta-analysis, latanoprost reduced IOP by 28%–31% from baseline, travoprost by 29%–31%, and bimatoprost by 28%–33%. Latanoprost and travoprost are selective prostanoid FP receptor agonists, and by binding to these receptors they exert their IOP-lowering effect [7, 8]. 

Bimatoprost is a prostamide, with a molecular mechanism of action not clearly understood [9, 10].

All these compounds decrease IOP by increasing aqueous outflow, mainly through the uveoscleral (unconventional) route [11].

PGAs appear to regulate matrix metalloproteinases (MMP) and tissue inhibitors of matrix metalloproteinases (TIMP) to modulate trabecular outflow resistance. MMPs are neutral zinc-dependent endoproteinases involved with normal and pathologic remodeling of extracellular matrix [12].

Increased expression of MMP-1, -3, -17, and -24 and TIMP-2, -3, -4 may lead to hydrolysis of collagen types I and III (MMP-1), collagen IV and fibronectin (MMP-2), and collagen types III, IV, fibronectin and laminin (MMP-3), resulting in the widening of the connective tissue-filled spaces among the ciliary muscle bundles and loss of trabecular meshwork (TM) extracellular matrix, hence increased outflow [11].

A new prostanoid receptor analogue in a preservative-free formulation, tafluprost, has been authorized for medical treatment of glaucoma and ocular hypertension.

Tafluprost differs from the other prostanoids available on the market for the presence of two fluorine atoms at the carbon-15 position, instead of the hydroxyl group present in latanoprost, travoprost, and bimatoprost [13]. Its affinity for the human prostanoid FP receptor is 12 times that of the carboxylic acid in latanoprost [14].

Glaucomatous patients often need to use topical therapy for many years, and, in order to promote compliance, adverse events and side effects should be minimized. Among these side effects, ocular surface disorders attributable to the drug itself or to drug preservatives are relatively common [15, 16].

The adverse influence of preservative-containing topical antiglaucoma medications on cells and tissues on the eye surface is well documented, both in vitro and in vivo studies [17, 18].

Benzalkonium chloride (BAK) is the most commonly used preservative in eye drops. It has already been found that this compound exerts cytotoxic (proapoptotic and pronecrotic) effects on the ocular surface and trabecular meshwork cells [19, 20].

Solutions preservative-free, containing lower BAK concentrations or alternative preservatives, were introduced into topical glaucoma therapy to minimize side effects. Among the widely used prostaglandin analogues, only tafluprost is actually available in a preservative-free formulation. A preservative-free solution of tafluprost showed reduced toxicity in human conjunctival epithelial cell lines when compared with preserved latanoprost, travoprost, and bimatoprost [21].

Despite several studies concerning its efficacy [22, 23], safety [24], and tolerability [25], the IOP lowering effect of tafluprost, as compared with the other prostaglandin analogues is not well established.

The purpose of this study was to assess the ocular hypotensive effect and the tolerability of tafluprost (0.0015%) in glaucoma patients previously treated with latanoprost (0.005%), travoprost (0.004%), or bimatoprost (0.03%) complaining for ocular discomfort. To the best of our knowledge, this is the first study comparing tafluprost to all the other PGAs commercially available.

## 2. Methods

This prospective clinical study was carried out at the Eye Clinic of the University of Milan, San Giuseppe Hospital, Milan, Italy, and it was approved by the local Ethical Committee conducted according to ICH/GCP guidelines. Patients of 18 years or older who fulfilled the eligibility criteria were recruited consecutively during routine visits and included in the study.

### 2.1. Inclusion Criteria

Patients with diagnosis of primary open-angle glaucoma based on the European Glaucoma Society Guidelines criteria, [5] treated with latanoprost, travoprost, or bimatoprost monotherapy for at least 3 months, complaining for ocular surface discomfort with baseline IOP less than 21 mmHg at all-time point with target IOP reached as set by the treating physician, were considered for the study.

### 2.2. Exclusion Criteria

Exclusion criteria included closed or barely open anterior chamber angle, or history of acute angle closure ocular trauma, history of ocular surgery, argon laser trabeculoplasty, ocular inflammation or infection occurring within 3 months before the baseline visit, neovascular glaucoma, history of refractive surgery, inability to adhere to the treatment and visit plan, other abnormal condition, or symptom preventing the patient from entering the trial, according to the investigator's judgment.

### 2.3. Study Plan

Patients treated with latanoprost, travoprost, or bimatoprost monotherapy for at least 3 months, IOP less than 21 mmHg, target IOP reached with ocular surface discomfort were enrolled in the study. At the baseline visit, a medical history was taken for all the subjects. All the subjects underwent a complete ophthalmic examination including anterior segment biomicroscopy and fundus examination, refraction and measurement of best-corrected visual acuity (BCVA) by means of Snellen chart. A masked operator (SR) measured IOP at 8 AM, 11 AM, 2 PM, 5 PM, and 8 PM by Goldmann applanation tonometry. If both eyes fulfilled the eligibility criteria, one eye was randomly selected for the study.

Patients were switched to tafluprost monotherapy without washout between the treatments. Assessment of IOP and tolerability were carried out 3-month after switching the therapy by the same masked operator (SR). The investigator could choose to have a nonscheduled safety visit between the scheduled visit. IOP mean value at each time point was calculated as a mean of three IOP diurnal curves carried out within three weeks at baseline and after 3 months.

### 2.4. Study Outcomes

The primary outcome was to compare the mean diurnal IOP from the daily curve after 3 months of tafluprost treatment compared to preservative prostaglandin analogue (latanoprost, travoprost, and bimatoprost). Secondary outcome was to compare the diurnal mean IOP of the tafluprost treatment after 3 months with each previous preservative PGAs treatment group as mean IOP and for each time point separately (8 AM, 11 AM, 2 PM, 5 PM, 8 PM).

### 2.5. Clinical Tolerability Assessments

Best-corrected visual acuity (BCVA) by means of Snellen chart, biomicroscopy, and ophthalmoscopy were recorded at the baseline visit and at each follow-up visit. Any kind of adverse event was recorded. Change of conjunctival hyperemia was recorded at the slit lamp using a standard scale ranging from 0 to 3 (where 0 is none, 1 is mild, 2 is moderate, and 3 is severe) with the help of a standardized photographic chart [26]. Superficial keratitis, defined as the presence of small circular epithelial erosion in the cornea, was also graded as none, mild, moderate, and severe (none: no staining; mild: rare stained erosion localized close to the lid margins; moderate: rare stained erosion localized in the 4 quadrants; diffuse: diffuse stained erosion involving the 4 quadrants).

### 2.6. Analysis

Data are presented as mean and 95% confidence intervals for continuous variables and frequencies for categorical variable. For the IOP recording, the mean values of 2 measurements at each time point were used in the calculations. If both eyes fulfilled the eligibility criteria, only one eye was randomly selected. An intent-to-treat approach was used to analyze the IOP variables. Categorical variables such as proportions and tolerability variable were analyzed using the Pearson chi-square test of Fisher exact test as appropriate. 

Formal sample size was calculated in order to assess the difference between treatments. Assuming Δ 2.3 mmHg (based on previously published data) [27] and using the formula:

(1) $$n=\frac{{\left[\Phi^{-1}\left(\alpha /2\right)+\Phi^{-1}\left(\beta \right)\right]}^{2}\sigma^{2}}{\Delta^{2}},$$

where  *α* = 0.05 and  1 − *β* = 0.80, a sample of 85 patients was needed to ensure a 95% chance of detecting a difference between the different treatments groups.

## 3. Results

A total of 91 patients were enrolled in this study, and 89 (mean age 64.7 ± 10.4) were included in the analysis (29 patients were under latanoprost, 28 under travoprost, and 32 under bimatoprost). Two patients were lost to followup without performing any follow-up visit and were excluded from the analysis. During the study we did not observe any adverse events.

### 3.1. Primary Outcome

Table 1 reports the mean daily IOP at baseline and after 3 months of treatment with tafluprost. After 3 months of treatment, mean daily IOP was not statistically different significantly compared with the baseline (+0.6 mmHg,  *P* > 0.05). No interaction between drug and treatment sequence was detected, indicating no carry-over effects between drugs.

**Figure 1:**
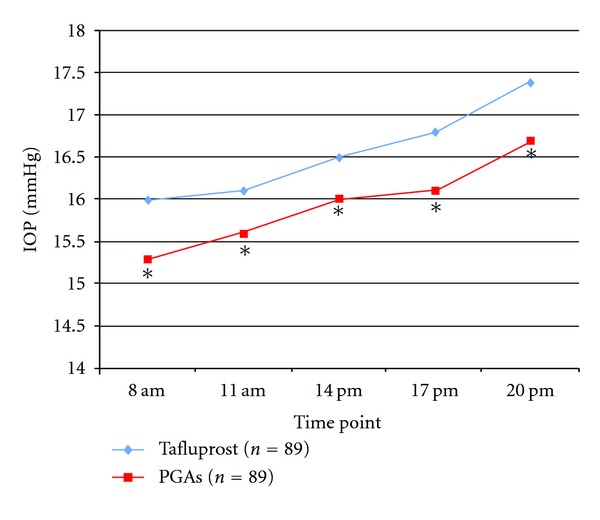
Graph showing IOP at each time point of the daily curve at baseline (PGAs) and after switching to tafluprost. *P > 0.05 from the comparison of tafluprost time point with the corresponding PGAs time point. (Curve of PGAs are prestudy, whereas curve of tafluprost is after dosing.)

Figure 1 reports the mean IOP value for each time point from the daily curve of tafluprost and PGAs treatment group. For each time point, separately, no differences were detected in mean IOP between the two groups (15.4 ± 2.4 versus 16 ± 2.3 at 8:00 AM, 15.6 ± 2.3 versus 16.1 ± 2.4 at 11 AM, 16.0 ± 2.4 versus 16.5 ± 2.4 at 2 PM, 16.1 ± 1.8 versus 16.8 ± 2.1 at 5 PM, 16.7 ± 2.6 versus 17.4 ± 1.8 at 8 PM, *P* > 0.05 for each time point).

 Globally, the incidence of local adverse events was similar in both treatments; mean conjunctival hyperemia was 1.2 ± 0.8 with preservative PGAs, and 1.0 ± 0.6 with tafluprost (*P* = 0.06), mean punctuate keratitis was 0.8 ± 0.6 with PGAs, and 0.8 ± 0.6 with tafluprost (*P* = 0.2).

In 75% of the patients, there was an improvement of ocular discomfort, in 21% of patients ocular discomfort was unchanged, and in 4% there was a worsening of ocular symptoms.

### 3.2. Secondary Outcome

**Figure 2:**
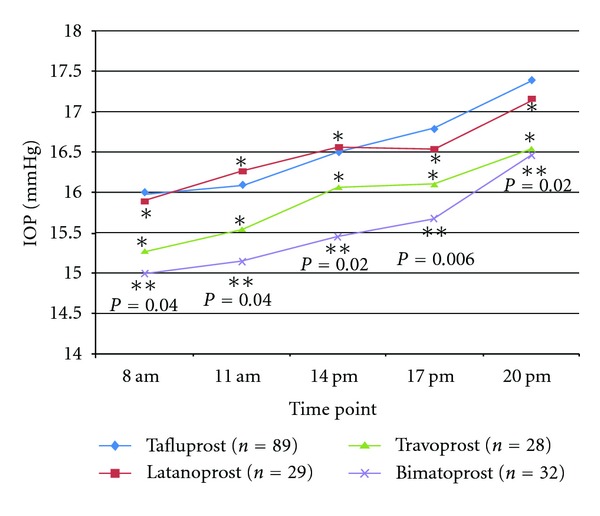
Graph showing IOP at each time point of the daily curve of each drug group. *P > 0.05 from the comparison of tafluprost time point with the corresponding latanoprost and travoprost time point. **Values from the comparison between tafluprost and bimatoprost of each time point of the daily curve. (Curve of latanoprost, travoprost, and bimatoprost are prestudy, whereas curve of tafluprost is after dosing.)

Figure 2 reports the comparison among the mean IOP value for each time point from the daily IOP curve of the four treatment groups. Comparing latanoprost to tafluprost treatment, no differences were found in mean IOP (16.5 ± 2.3 versus 16.6 ± 2.0, *P* = 0.85) and at each time point (15.9 ± 2.6 versus 16.0 ± 2.3 at 8:00 AM, 15.6 ± 2.3 versus 16.1 ± 2.4 at 11 AM, 16.0 ± 2.4 versus 16.5 ± 2.4 at 2 PM, 16.1 ± 1.8 versus 16.8 ± 2.1 at 5 PM, 16.7 ± 2.6 versus 17.4 ± 1.8 at 8 PM, *P* > 0.05 for each time point).

No differences were found in conjunctival hyperemia and punctuate keratitis between the two groups (*P* = 0.8 and *P* = 0.9, resp.).

Comparing travoprost to tafluprost treatment, no differences were found in mean IOP (15.9 ± 2.5 versus 16.6 ± 2.0, *P* = 0.15) and at each time point (15.3 ± 2.5 versus 16.0 ± 2.3 at 8:00 AM, 15.5 ± 2.5 versus 16.1 ± 2.8 at 11 AM, 16.1 ± 2.8 versus 16.5 ± 2.4 at 2 PM, 16.1 ± 1.9 versus 16.8 ± 2.1 at 5 PM, 16.5 ± 2.7 versus 17.4 ± 1.8 at 8 PM, *P* > 0.05 for each time point).

No differences were found in conjunctival hyperemia and punctuate keratitis between the two groups (*P* = 0.1 and *P* = 0.3, resp.).

Comparing bimatoprost to tafluprost treatment a statistically significant difference was found in mean IOP (15.6 ± 1.8 versus  16.6 ± 2.0,  *P* = 0.01) with a mean increase of 1 mmHg of IOP.

A significant difference in IOP was found at each time point (15.0 ± 2.4 versus 16.0 ± 2.3 at 8:00 AM, 15.1 ± 1.7 versus 16.1 ± 2.4 at 11 AM, 15.5 ± 2.0 versus 16.5 ± 2.4 at 2 PM, 15.7 ± 1.6 versus 16.8 ± 2.1 at 5 PM, 16.5 ± 2.4 versus 17.4 ± 1.8 at 8 PM, *P* < 0.05 for each time point).

The severity of conjunctival hyperemia and punctuate keratitis was higher with bimatoprost compared to tafluprost (1.3 ± 0.9 versus  1.0 ± 0.6, *P* = 0.02 for conjunctival hyperemia and 0.9 ± 0.7 versus 0.7 ± 0.6, *P* = 0.04 for punctuate keratitis).

## 4. Discussion

This prospective study aims to compare the IOP lowering effect of tafluprost with other PGAs in patients with glaucoma, with signs and symptoms of ocular discomfort. Published studies suggest that the IOP lowering effect of tafluprost is similar to other PGAs with a better local safety profile due to the absence of BAK [14, 22, 24, 25]. Several study has been published about safety and efficacy profile of tafluprost in vitro [19, 21, 23] and in animal models [7, 12, 14, 22, 25, 26], but few studies analyse the IOP lowering effect in a clinical setting in patients with glaucoma and ocular hypertension [28–32].

The primary outcome of this study was to compare the IOP lowering effect of tafluprost with all other BAK preserved PGAs.

After 3 months of treatment the mean daily IOP was not statistically significant different compared with preserved PGAs.

The comparison among the single PGAs and tafluprost showed a comparable efficacy between latanoprost and tafluprost (*P* = 0.85) and between travoprost and tafluprost (*P* = 0.15). The mean daily IOP and the IOP at any time point did not show any significant difference.

The bimatoprost group showed a statistically lower mean IOP, as compared to tafluprost (*P* = 0.01), and the IOP lowering effect was statistically greater at any time point of the diurnal IOP curve.

The analysis of the ocular surface disorders showed no statistically difference between latanoprost and tafluprost and between travoprost and tafluprost. Bimatoprost showed to induced a slightly higher conjunctival hyperemia and punctuate keratitis compared to tafluprost (*P* < 0.05).

Only few published studies compared tafluprost with other PGAs [28–32]. Most of them compare tafluprost with latanoprost [30, 31, 33]. There is only one study comparing tafluprost with travoprost [28] and none with bimatoprost.

In a recently published study [28] comparing efficacy of tafluprost with travoprost in patients with glaucoma or ocular hypertension, the authors found a statistically significant greater IOP lowering efficacy with travoprost compared to tafluprost (16.9 mmHg versus 17.5 mmHg, resp., *P* = 0.01), with a similar safety profile for both the treatments.

Our study involved only glaucoma patients, whereas glaucoma and ocular hypertension patients were enrolled in Schnober study [34]. The different population involved could explain the different results.

In the study by Mochizuki et al. [28], the comparison between tafluprost and latanoprost in a group of healthy volunteers showed a statistically greater IOP reduction with tafluprost. In this study tafluprost was associated with a higher rate of conjunctival hyperemia.

Uusitalo et al. [29] investigated the efficacy of tafluprost in patients treated with latanoprost exhibiting ocular surface side effects. 158 patients were switched from latanoprost to tafluprost. After 12 weeks, tafluprost maintained the IOP at the same value as latanoprost baseline (16.4 ± 2.7 mmHg versus 16.8 ± 2.5 mmHg, resp.) with a decrease in subjective symptoms and objective signs.

Another study by Traverso et al. [30] reports the IOP lowering efficacy of tafluprost in patients previously treated with latanoprost. A preservative formulation of tafluprost was used in this trial. The lowering effect at 24 months was −7.1 mmHg and −7.7 mmHg in tafluprost and latanoprost group, respectively. Although a slightly larger IOP lowering effect of latanoprost, the ANOVA test showed a noninferiority of tafluprost to latanoprost.

Efficacy of tafluprost was compared with latanoprost in glaucoma and ocular hypertension patients in a recent published randomized, controlled study [31]. The mean IOP reduction was −9.7/−3.3 mmHg in the tafluprost group and −8.8 ± 4.3 mmHg in the latanoprost group, respectively. The difference was not significantly different, and the author concluded that both tafluprost and latanoprost have a comparable effect on IOP lowering effect.

In agreement with published studies conducted with glaucoma patients, we found a similar efficacy of tafluprost compared to latanoprost. In the Mochizuki study, healthy patients were enrolled. The different results of this study are likely to depend on the different population.

Hommer [31] recently published a large series of 544 patients with glaucoma or ocular hypertension treated with preserved eye drops and poor IOP control or poor local tolerance. In these patients previous medications were changed with tafluprost. Previous therapy was monotherapy, fixed and nonfixed combination. 45 patients were naïve to treatment. In this heterogeneous group of patients tafluprost showed to provide a further IOP decrease with an overall reduction of IOP in all patients from 19.4 ± 5.0 mmHg at baseline to 15.3 ± 3.5 mmHg at 12 weeks. Author found an improvement in signs and symptoms related to ocular surface inflammation after switching with tafluprost.

Unlike the data from Hommer study, we did not found any additional IOP lowering effect of tafluprost compared to other medications. This could be partially explained by the heterogeneous population enrolled in this study.

According to other studies  [8, 28, 32]   bimatoprost showed to be the most effective prostanoid analogue in this series of patients. 

The decrease in ocular inflammation signs and symptoms has been detected in this study only comparing tafluprost with bimatoprost. The safe profile of the tafluprost showed in our study is consistent with published data about in vivo and in vitro safety of the unpreserved eye drops [19–22, 25].

The lack of the washout between treatments may be a limitation of the study. Nevertheless, the IOP lowering effect was evaluated 3 months after the switch of the therapy: this should be considered a sufficient time for the complete wash out of previous treatments. Other limitation is the short followup that makes challenging to identify any long-term efficacy and safety differences between different PGAs. The sample size of the study was calculated for the first outcome and is underestimated for the comparison between each group of PGAs and tafluprost. Prospective, randomized, possibly multicenter study with a larger sample size it will be needed to better clarify the long-term efficacy and safety profile of this new unpreserved PGA.

In conclusion, this is the first study comparing the IOP lowering efficacy of the all commercially available BAK preserved PGAs (latanoprost 0.005%, travoprost 0.004%, bimatoprost 0.03%) with the new unpreserved PGA (tafluprost 0.0015%). Tafluprost demonstrated a IOP lowering effect similar to others PGAs and after 3 months of therapy with tafluprost the mean IOP showed no difference between tafluprost and BAK preserved PGAs.

Tafluprost showed, towards other PGAs, comparable efficacy and a safe profile. Furthermore in this study bimatoprost seems to provide a statistically significant additional IOP lowering effect compared to patients treated with tafluprost.

## Figures and Tables

**Table 1:** Mean daily IOP at baseline and after 3 months of treatment with tafluprost.

	Baseline IOP	After 3 months of tafluprost	Mean IOP change	Mean % IOP change	*P* value
Latanoprost, travoprost, or bimatoprost treatment	16 ± 2.1 mmHg	16.6 ± 2.0 mmHg	+0.6 mmHg	+3.75%	>0.05
